# Exenatide for obesity in children and adolescents: Systematic review and meta-analysis

**DOI:** 10.3389/fphar.2024.1290184

**Published:** 2024-04-03

**Authors:** Bin Chen, Zhuan Zou, Xiaoyan Zhang, Dongqiong Xiao, Xihong Li

**Affiliations:** ^1^ Department of Pediatrics, West China Second University Hospital, Sichuan University, Chengdu, China; ^2^ Key Laboratory of Birth Defects and Related Diseases of Women and Children, Sichuan University, Chengdu, China

**Keywords:** exenatide, obesity, efficacy, child, meta-analysis

## Abstract

**Objectives:** There is no curative treatment for childhood obesity. We aim to synthesize published Randomized Controlled Trials (RCTs) evidence on the efficacy of exenatide in obese children and adolescents.

**Methods:** We conducted a comprehensive search and analysis of relevant studies in popular databases such as PubMed, Web of Science, Embase, and Cochrane Library. Our focus was on RCTs that examined the effectiveness of exenatide for treating obesity in children. We primarily assessed changes in body weight, body mass index (BMI), fasting plasma glucose (FPG), or HbA1c levels. Additionally, we considered any adverse events reported during the treatment period, with particular attention to hypoglycemia. To evaluate the quality of RCTs included in our study, we employed the Cochrane bias assessment tool.

**Results:** Five studies met the inclusion criteria. A group of 100 children were assigned to receive treatment with exenatide. Compared with controls, exenatide therapy reduced body weight and BMI by −0.6% (95% CI −0.93, −0.27), −1.11% (95% CI −1.91, −0.31), respectively. Undesirable consequences encompass gastrointestinal symptoms, with the majority of instances being characterized by mild severity.

**Conclusion:** Exenatide demonstrates efficacy in the treatment of pediatric and adolescent obesity.

**Systematic Review Registration:** PROSPERO https://www.crd.york.ac.uk/prospero/display_record.php?RecordID=413706

## 1 Introduction

In the last 5 decades or so, the prevalence of obesity has increased worldwide, according to the World Health Organization (WHO), over 650 million adults are obese, three times more than the rate in 1975, and another 1.3 billion people are overweight worldwide (Carreira et al., 2022). The COVID-19 pandemic made the situation more serious, with more than 50% of individuals with obesity reporting increased weight during the lockdown (Sideli et al., 2021). Basically, the rate of increase in childhood obesity has been higher than that in adults and it is expected that there will be 108 million obese young people worldwide (Afshin et al., 2017). Excessive body weight frequently leads to long-term health complications. Children can develop diabetes mellitus (Suárez et al., 2023), cardiovascular disease (Singh et al., 2013), many cancers (Lauby-Secretan et al., 2016; Afshin et al., 2017), and depression (Oz and Kıvrak, 2023). The health problem of obesity severely affects the quality of life and presents a more serious health threat. Hence, it is crucial to ensure timely and consistent weight reduction during childhood.

The lack of weight-loss treatments goes against the idea of worldwide health equality. The initial approach to addressing childhood obesity entails making changes to one’s lifestyle. Which means children need help to choose better foods and find ways to exercise more. Indeed, long-term weight loss maintenance through lifestyle changes is elusive for most obese children. This option can be further advanced to drug treatment, and among the most hopeful categories of medications is the agonist for glucagon-like peptide-1 receptor agonist (GLP-1RA), which reduces appetite through activation of GLP-1 receptors in the hypothalamus and slows down the rate at which a person’s stomach empties, promotes satiety, and reduces food intake (Bojanowska, 2005; Alhadeff and Grill, 2014; Burcelin and Gourdy, 2017). Besides weight loss, GLP-1 RA has been shown to improve glucose control by the glucose-dependent increased in postprandial insulin secretion, and inhibition of glucagon secretion improves many obesity-associated risk factors and complications, such as impaired glucose tolerance, reduced insulin sensitivity, elevated blood pressure, impaired vascular function, and increased inflammatory response (Koska et al., 2010; Okerson et al., 2010; Kelly et al., 2013).

Exenatide, a member of the GLP-1RA class, was initially developed and subsequently granted approval for managing Type 2 diabetes (T2DM) in adults and have also been shown to reduce weight in adults with obesity (Vilsbøll et al., 2012). The Core DURATION trial series was conducted from 2008 to 2013, the duration of exenatide treatment ranges from 24 to 30 weeks, and the average weight loss of patients during treatment ranged from 2.0 to 3.7 kg (Drucker et al., 2008; Bergenstal et al., 2010; Diamant et al., 2010; Blevins et al., 2011; Russell-Jones et al., 2012; Buse et al., 2013). Multiple meta-analyses of RCTs in adults with T2DM and/or obesity have shown benefits of exenatide for glycemic control and weight loss (Vilsbøll et al., 2012; Su et al., 2016; Monami et al., 2017; Waldrop et al., 2018). The use of liraglutide, a GLP-1 agonist, has been approved by the FDA for treating children who have T2DM(Bacha, 2019). Additional evidence is required to support the utilization of exenatide in pediatric patients.

This study aims to consolidate the findings from various randomized controlled trials (RCTs) and conduct a meta-analysis to evaluate the effectiveness and safety of exenatide in managing obesity among children.

## 2 Materials and methods

The review of existing literature was conducted in accordance with the guidelines provided by PRISMA, which are designed for systematic reviews and meta-analyses (Moher et al., 2009).

### 2.1 Eligibility criteria

We devised a framework called “PICOS” (Patient, Intervention, Comparison, Outcome and Study design) to establish the criteria for eligibility: 1. Population: Pediatric patients (≤18 years) with a diagnosis of obesity as defined by the study authors; 2. Intervention: exenatide as the intervention group. Medications that affect blood sugar or other parameters of metabolic syndrome should not be taken within 3 months prior to screening; 3. Comparison: control group receiving either placebo or standard treatment. Both the intervention and control groups take exenatide as an adjunct to diet and exercise; 4. Outcome: clinical trials meeting the eligibility criteria were required to document results for a minimum of one among the subsequent endpoints: HbA1c, FDP, body weight or BMI change; 5. Study design: RCTs. The exclusion criteria encompassed studies that were not randomized controlled trials (non-RCTs), as well as systematic reviews, meta-analyses, narrative reviews, editorials, abstracts, reports, and case series.

### 2.2 Information sources and search strategy

PubMed, Web of Science, Embase, and Cochrane Library were searched up until 6 March 2023, with no cut-off criteria for publication date, using the following search terms: (“Exenatide”) AND (“Pediatric Obesity”) AND (“Children”) AND (“Adolescent”). Keywords and Mesh/Emtree thesauri were applied to establish search strategies for each database. Two different investigators (C.B. and Z.X.Y.) independently evaluated the search results. A search strategy example is depicted in Supplementary Figure S1.

### 2.3 Study selection

After exclusion of duplicates, two reviewers (C.B. and Z.C.) independently screened the title and abstract. In the process of title and abstract screening, we carefully chose articles that seemed to fulfill the eligibility criteria. In case of any discrepancies between the two reviewers, they were resolved through discussion or referred to the corresponding author (L.X.H.) for final judgment.

### 2.4 Data extraction and quality assessment

The provided RCTs were analyzed to obtain the subsequent details: primary author, year of publication, number of participants, demographic characteristics of the study population, and geographical location, blood glucose (i.e., HbA1c in percent and FPG in mg/dL) and weight measures (i.e., weight in kilogram, BMI in kg/m^2^) with corresponding standard deviations (SDs) at baseline and follow-up, change-from-baseline blood glucose and weight outcomes with corresponding SDs, difference in change-from-baseline blood glucose and weight outcomes with corresponding confidence intervals (CIs) or *p* values, target drug dose and follow-up duration, the adverse events, and incidence of hypoglycemic episodes. Two reviewers (Z.X.Y. and X.D.Q.) extracted data from each trials independently. Missing SDs at follow-up were imputed by reported SDs at baseline, or *vice versa* (Cumpston et al., 2019). The risk of bias assessment for the included RCTs was conducted by two reviewers (C.B. and Z.X.Y.) utilizing the Cochrane Collaboration tool (Sterne et al., 2019). Disputes were settled through deliberation or assessed by the corresponding author (L.X.H.).

### 2.5 Meta-analysis

The effects of exenatide on difference Among change-from-baseline body weight, BMI, HbA1c, FPG outcomes were summarized by employing (i) statistical models with random effects and weighted variances to account for variations in individual study findings; (ii) statistical models with fixed effects and weighted variances assuming consistent effect estimates across studies. The findings of meta-analyses were presented in the form of mean differences (M.D.) for continuous outcomes and relative risks (R.R.) for dichotomous outcomes. The Hartung-Knapp technique was utilized to update the 95% confidence intervals (C.I.s) for the effects. The inconsistency index (I^2^) was used to assess heterogeneity between studies, with studies having an I^2^ value greater than 50% considered to have significant heterogeneity. Funnel plots and Egger’s regression tests were employed to examine asymmetry in the effect estimates at the study level. Sensitivity analyses using leave-one-out methods were conducted to test the robustness of the meta-analysis models. Subgroup analysis was performed to explore potential sources of heterogeneity. Statistical analyses were carried out using STATA software from Stata-Corp LLC located in College Station, TX, United States of America.

## 3 Results

### 3.1 Summary of study characteristics

**FIGURE 1:**
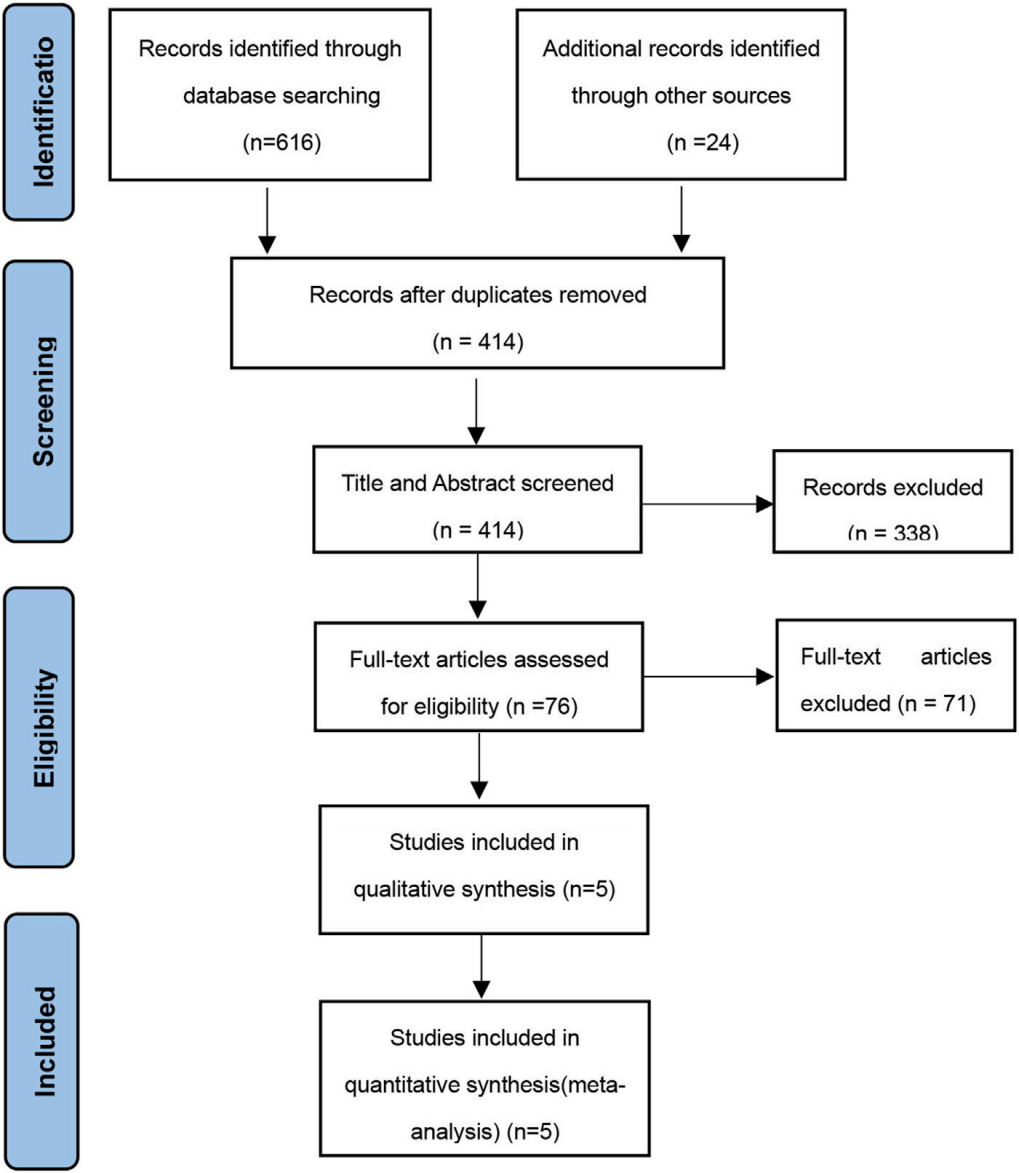
Flow chart of the literature search.

The study selection process is visually represented in Figure 1 through a flow diagram. Five RCTs met the criteria for inclusion in the systematic review (Kelly et al., 2012; Kelly et al., 2013; Weghuber et al., 2020; Roth et al., 2021; Fox et al., 2022). The key features of these studies are outlined in Table 1. Only one study was conducted in Europe (Weghuber et al., 2020), and the others in the United States. All the studies were published from 2012 to 2022. Mean ages of children ranged from 12.7 to 16.9 years old, treatment durations from 11 to 52 weeks, and study sizes from 11 to 66 children. Across all studies, 100 children were allocated to exenatide therapy. Treatment dose of five mcg, twice per day in two studies (EX10BID) (Kelly et al., 2012; Kelly et al., 2013), 2 mg once-weekly in three studies (EX2QW) (Weghuber et al., 2020; Roth et al., 2021; Fox et al., 2022). The participants in one study were patients with intracranial tumors after hypothalamic injury or children with hypothalamic obesity (Weghuber et al., 2020). One clinical trial utilized an open-label, crossover design (Kelly et al., 2012), while the remaining studies followed a double-blind, randomized design with placebo control. We expect positive changes in the health status of pediatric obese patients treated with exenatide in these anticipate observing a decrease in the occurrence of unfavorable incidents across five conducted studies, providing efficacy and safety results.

**TABLE 1:** Characteristics of the included randomized control trials of exenatide in children.

Study	Mean age (years)	N total (N treated)	Baseline HbA1c (%)	Baseline BMI (kg/m^2^)	Baseline glucose (mg/dL)	Baseline weight (kg)	Treatment dose	Treatment duration (weeks)	Outcome indicator
Kelly2012	12.7	11 (11)^a^	NR	36.7	75.4	93.8	0.01 mg/day	13	B, C, D
Kelly2013	15.2	22 (11)	5.26	42.5	79.6	124	0.01 mg/day	13	A, B, C, D
Weghuber2020	14.5	44 (22)	NR	36	95.1	106.2	2 mg/week	24	B, C, D
Roth2021	16.9	42 (23)	NR	37.3	NR	98.9	2 mg/week	36	A, D
Fox2022	16.0	66 (33)	5.2	39.4	76.7	108.5	2 mg/week	52	A, B, C, D

^a^ Cross-over study design; N: number; BMI, body mass index; HbA_1c_: hemoglobin A_1c_; NR: not report; A: HbA1c; B: fasting plasma glucose; C: body weight; D: body mass index.

**FIGURE 2:**
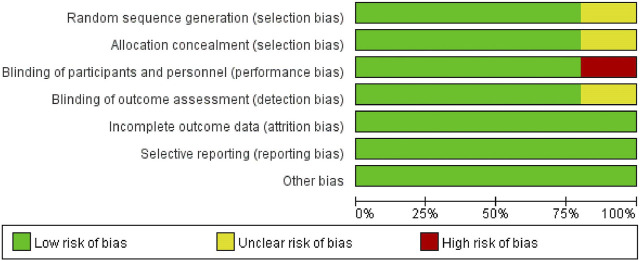
Risk of bias graph.

**FIGURE 3:**
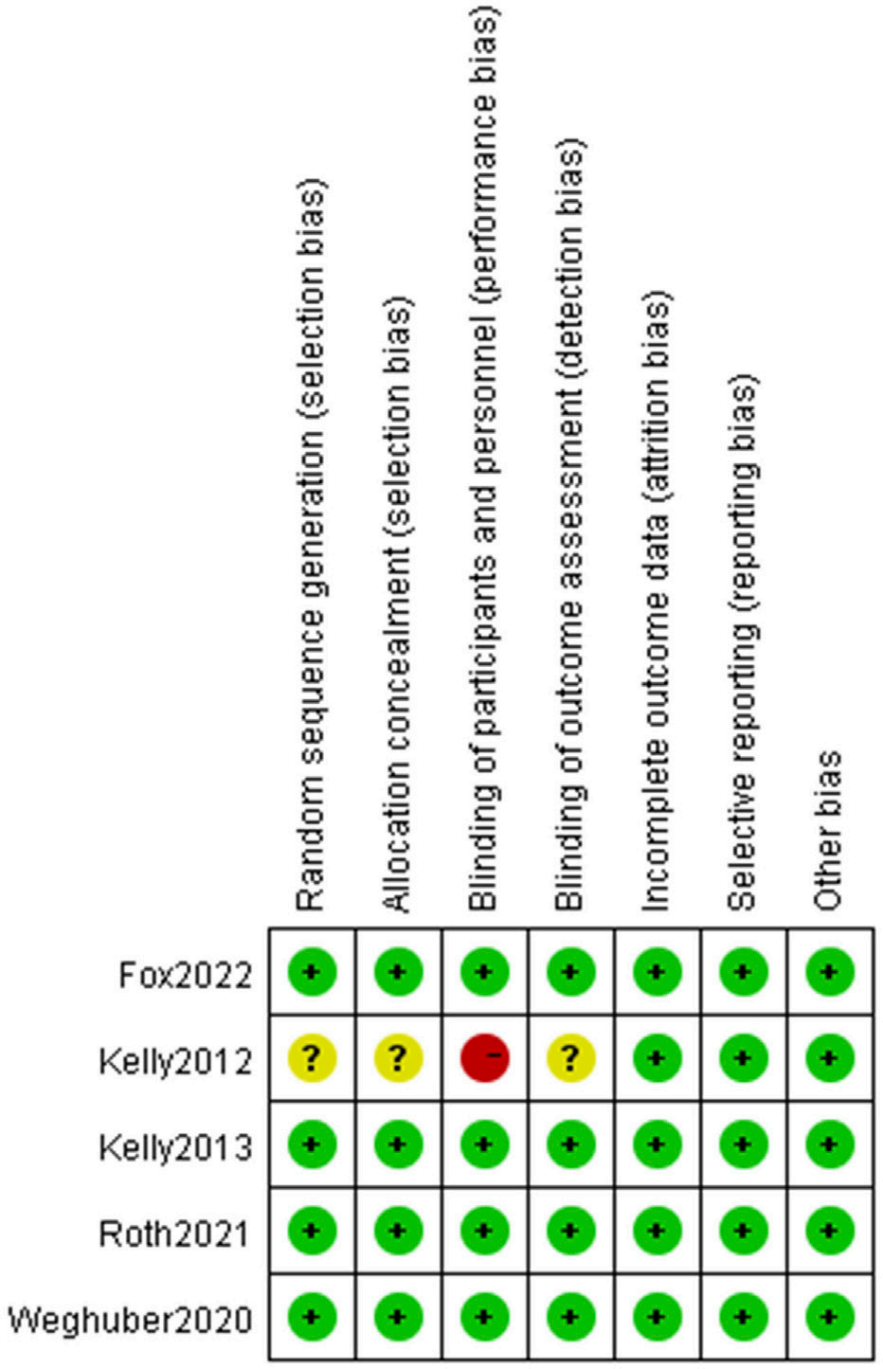
Risk of bias summary.

The assessment of potential bias in the studies included can be observed in Figure 2 and Figure 3. The studies conducted by Kelly (Kelly et al., 2012) was rated as poor quality due to open-label, crossover designing. While it was presumed that the remaining studies possessed a high level of quality.

### 3.2 Effect of exenatide on primary outcomes

**FIGURE 4:**
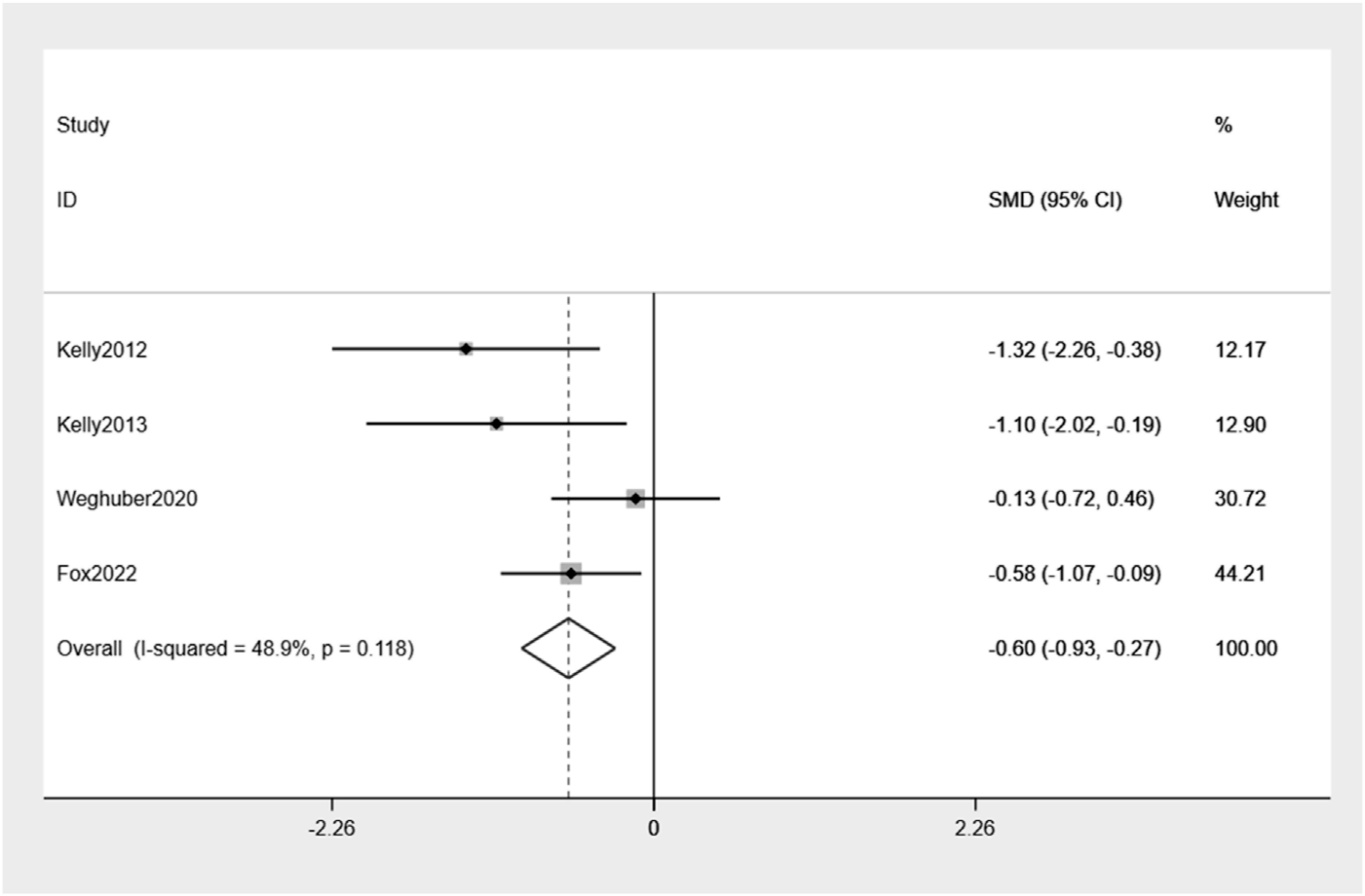
Forest plot of body weight reduction between exenatide group and control group.

The examination of the four randomized controlled trials indicated a slight reduction in body weight, amounting to −0.60% (95% CI: −0.93, −0.27), compared to placebo (Figure 4). Mild heterogeneity between the studies was observed (I^2^ = 48.9%).

**FIGURE 5:**
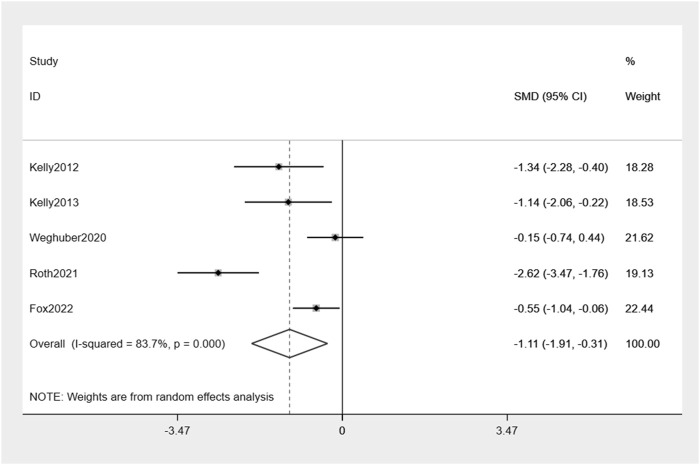
Forest plot of induction of BMI change between exenatide group and control group.

The analysis combining the results of all five studies revealed a significant decrease in BMI by −1.11% (95% CI: -1.91, −0.31) compared to placebo (Figure 5). There was moderate heterogeneity in effect estimates among the studies (I^2^ = 83.7%).

**FIGURE 6:**
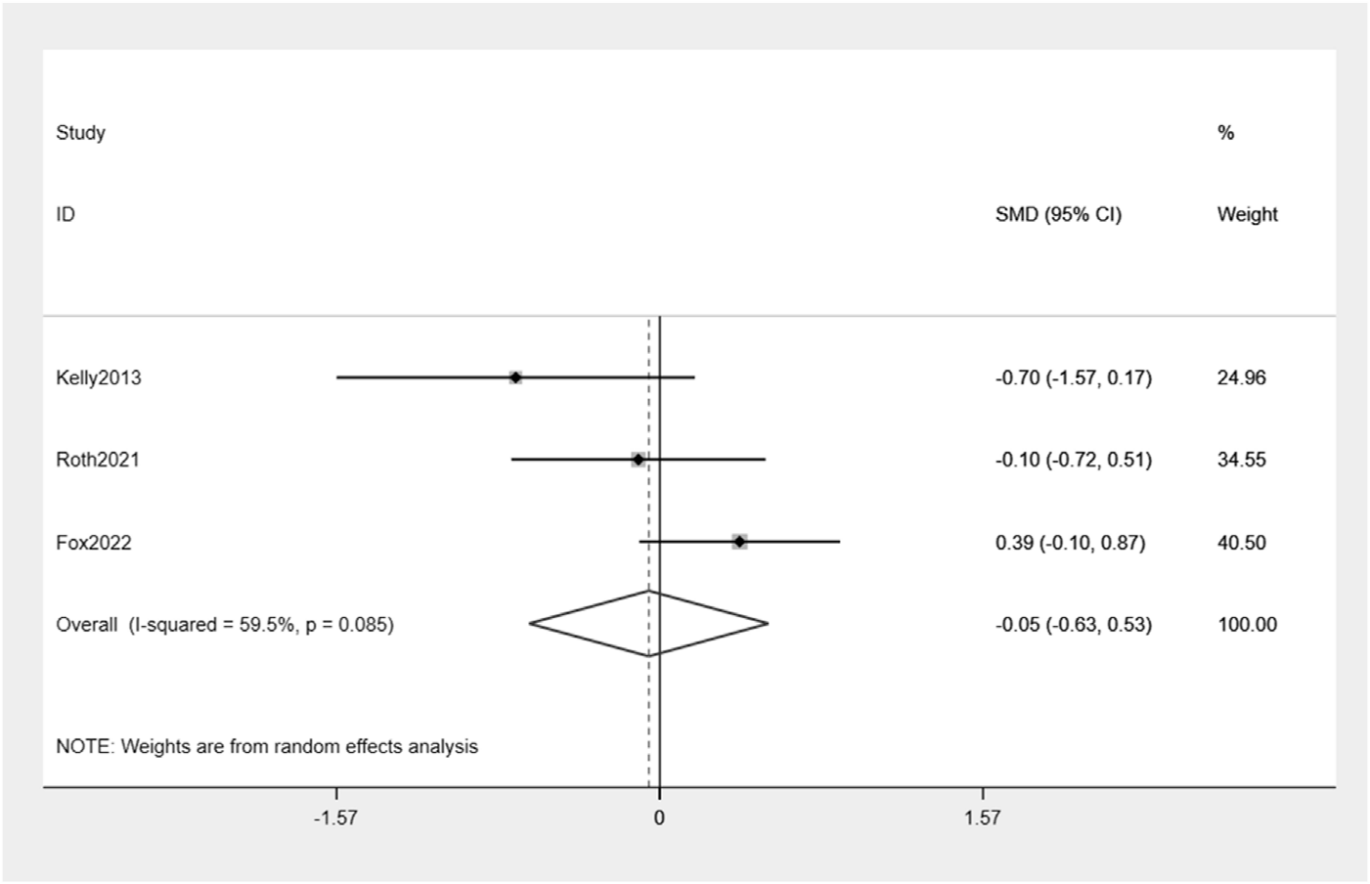
Forest plot of HbA1c change between exenatide group and control group.

Exenatide treatment did not result in a significant reduction in HbA1c levels compared to placebo (−0.05%, 95% CI: -0.63, 0.53) (Figure 6). Moderate heterogeneity between the studies was observed (I^2^ = 59.5%).

**FIGURE 7:**
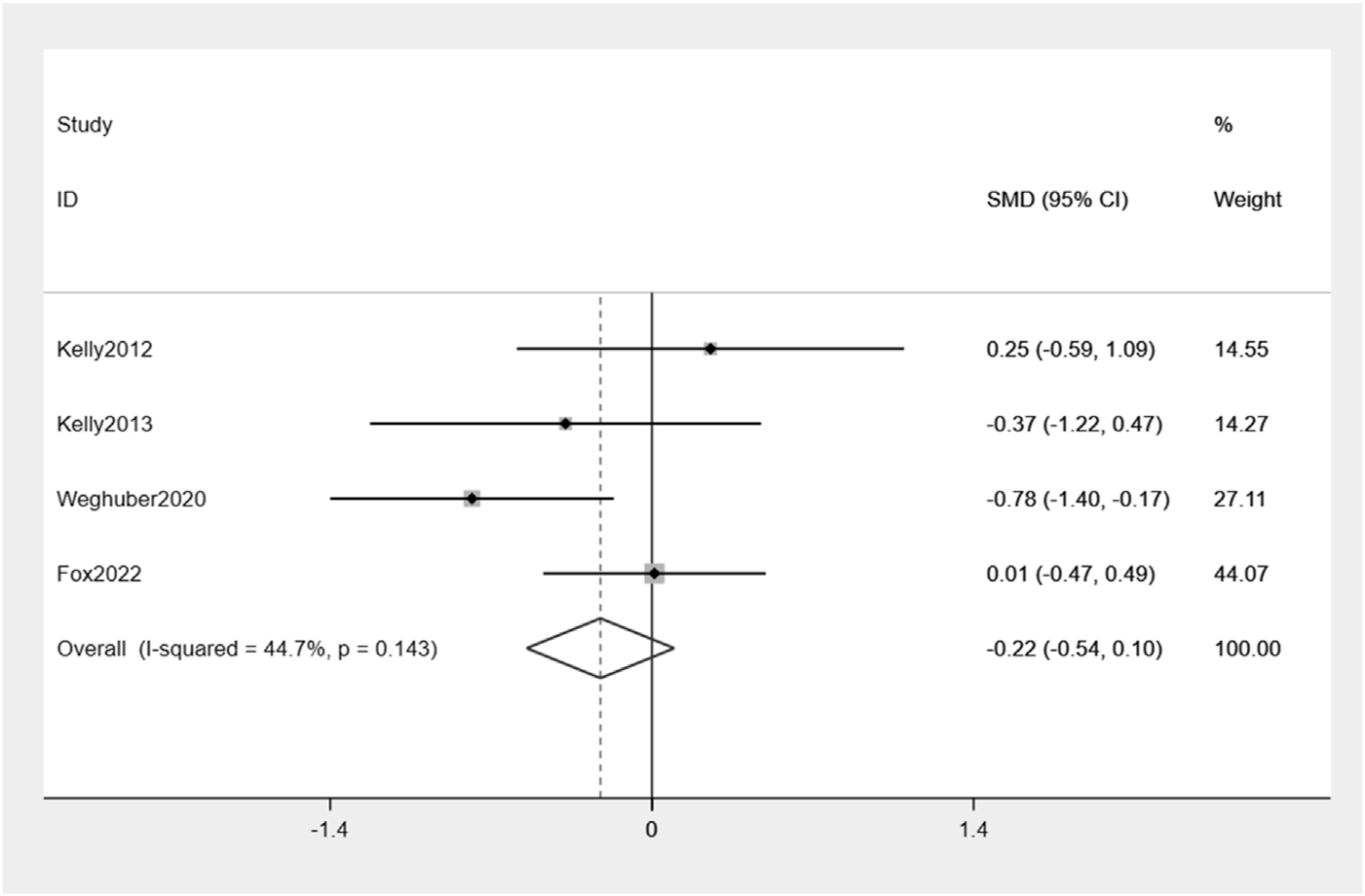
Forest plot of FPG change between exenatide group and control group.

Similarly, exenatide exhibits no significant reduction in FPG when compared to the placebo group: −0.22% (95% CI: −0.54, 0.10) (Figure 7). There was a moderate level of variability in effect estimates across the studies (I^2^ = 44.7%).

### 3.3 Sensitivity analyses

Leave-one-out analyses are depicted in Supplementary Figures S2, S3. For BMI and HbA1c, exclusion of any single arm did not significantly alter heterogeneity among studies.

### 3.4 Subgroup analysis of BMI

We used BMI for subgroup analysis among the participants based on baseline body weight, baseline BMI, obesity type and treatment does.

The results showed that participants with baseline body weight<100 kg (34 children) were associated with a greater decrease in percentage of BMI change by −1.99% (95% CI: -3.24, −0.74) compared to participants with baseline body weight>100 kg (67 children): -0.52% (95% CI: −0.99, −0.06) (Supplementary Figure S4).

Participants with baseline BMI>38 kg/m^2^ (45 children)were associated with a slight decrease in percentage of BMI change by −0.71% (95% CI: -1.23, −0.20), while there was no significant reduction in BMI among participants with baseline BMI<38 kg/m^2^ (56 children): -1.34% (95% CI: -2.86, 0.17) (Supplementary Figure S5).

Children with hypothalamic obesity (23 children) were associated with a greater decrease in percentage of BMI change by −2.62% (95% CI: -3.47, −1.76). While an overall reduction of BMI in obesity children without hypothalamic (78 children) injure by −0.68% (95% CI: -1.17, −0.19) (Supplementary Figure S6).

Participants receiving treatment doses of EX10BID (23 children) were associated with a greater decrease in the percentage of BMI change by −1.24% (95% CI: -1.89, −0.58). While participants who used with EX2QW (78 children) did not show a significant decrease in BMI: -1.06% (95% CI: -2.28, 0.16) (Supplementary Figure S7).

### 3.5 Subgroup analysis of body weight

We used body weight for subgroup analysis among the participants based on baseline body weight, baseline BMI and treatment does.

The results showed that participants with baseline body weight<100 kg (44 children) were associated with a greater decrease in percentage of body weight change by −1.32% (95% CI: -2.26, −0.38) compared to participants with baseline body weight>100 kg (34 children) by −0.50% (95% CI: −0.85, −0.15) (Supplementary Figure S8).

Participants with baseline BMI>38 kg/m^2^ (45 children) were associated with a greater decrease in percentage of body weight change by −0.70% (95% CI: -1.13, −0.26), while participants with baseline BMI<38 kg/m^2^ (33 children) does not decrease in body weight: -0.47% (95% CI: -0.97, 0.04) (Supplementary Figure S9).

Participants receiving treatment doses of EX10BID (23 children) showed a greater decrease in the percentage of body weight change by −1.21% (95% CI: -1.86, −0.55). While participants who used with EX2QW (55 children) exhibited a smaller decrease in body weight by −0.39% (95% CI: -0.77, −0.02) (Supplementary Figure S10).

### 3.6 Reported adverse events

All five studies provided data on the adverse events associated with exenatide therapy. Gastrointestinal symptoms, such as nausea, vomiting, and diarrhea, were identified as the most prevalent side effects. The majority of these instances were classified as mild in intensity. Notably, there was no statistically significant disparity observed in the occurrence rate between individuals receiving exenatide treatment and those administered a placebo. Only one serious adverse event in the exenatide treatment about a participant was hospitalized for chemical dependency treatment after a drug (not related to the study) and alcohol overdose (Fox et al., 2022). And none of the five studies documented any instances of hypoglycemia.

## 4 Discussion

In this systematic review and meta-analysis, the evidence from five RCTs was combined to analyze the efficacy and safety of exenatide in obese children Compared with previous systematic reviews (Su et al., 2016; Tsapas et al., 2021; Zhang et al., 2021; Guo et al., 2022), this research examined the effects of exenatide on children who are overweight or obese but do not have diabetes.

Currently, few drugs are approved for childhood obesity in children, and the evidence for treatment options is inadequate (Cornejo-Estrada et al., 2023b). GLP-1 agonists have been authorized for managing adult T2DM and obesity, while liraglutide has obtained approval for treating children with T2DM and obesity. Despite the structural similarity between exenatide and liraglutide, variations in their pharmacokinetic and pharmacodynamic properties lead to distinct effects on reducing hyperglycemia and weight. Liraglutide, a GLP-1 receptor agonist, exhibits a remarkable similarity of over 97% in its amino acid sequence to human GLP-1. It possesses an extended half-life of approximately 13 h. Conversely, exenatide has a shorter half-life compared to liraglutide, being only half as long, and demonstrates only 53% homology in its amino acid sequence with human GLP-1 (Lund et al., 2014). Exenatide and liraglutide have been demonstrated to be effective in numerous clinical studies for patients with T2DM(Bergenstal et al., 2010; Hall et al., 2013). In a head-to-head trial, patients who received liraglutide demonstrated greater weight loss (3.9 vs. 2.7 kg) compared to individuals treated with exenatide (Buse et al., 2013). However, exenatide and liraglutide have been demonstrated to be similar effective in promoting weight loss in numerous studies (Scott et al., 2013; McAdam-Marx et al., 2016; Saunders et al., 2016; Fadini et al., 2019). The current research extensively examined the use of exenatide in overweight children, offering a comprehensive analysis. The results demonstrate that treatment with exenatide is associated with a reduction in body weight and BMI among pediatric patients. These findings are consistent with those of Gou H et al., whose study also showed that liraglutide use was associated with lower body weight and BMI in pediatric patients (Gou et al., 2023). The results of another meta-analysis confirmed that there was no clinically significant difference in weight loss and BMI between liraglutide use and pediatric patients. However, it should be noted that only three studies were included in the systematic review, all of which were conducted in the United States (Cornejo-Estrada et al., 2023a). In a *post hoc* analysis of the randomized, placebo-controlled, SCALE Teens trial investigating predictive characteristics of liraglutide *versus* placebo for achieving a reduction in BMI of ≥5% and ≥10% after 56 weeks (Bensignor et al., 2023), it was found that liraglutide was more effective than placebo in reducing BMI by ≥ 5% and ≥10%. In the randomized, double-blind trial involving adolescents with obesity, the use of liraglutide in combination with lifestyle therapy resulted in a significantly greater reduction in BMI and body weight compared to placebo combined with lifestyle therapy. (Kelly et al., 2020).

In relation to effectiveness, there was no observed decrease in HbA1c and FDP levels among obese children treated with exenatide. Overall, exenatide reduced body weight by −0.6% and reduced BMI by −1.11%; however, higher mean baseline values of BMI corresponded to larger treatment effects on BMI, but lower mean baseline values of body weight corresponded to larger treatment effects on body weight change. Furthermore, the effect of both body weight loss was much larger in children treaded with EX10BID than EX2QW (−1.21% vs. −0.39%), and the similar effect on BMI (−1.24% vs. −1.06%). Interestingly, effects of exenatide on BMI were much more obvious among children with hypothalamic obesity than children without hypothalamic obesity (-2.62% vs. −0.68%).

In relation to the aspect of safety, children exhibited good tolerance towards exenatide therapy. Only few adverse events reported across the RCTs. And no instances of hypoglycemia have been documented. The prevalent adverse effects observed were gastrointestinal symptoms, particularly nausea, vomiting, abdominal discomfort, and diarrhea. These manifestations frequently resolved spontaneously without any intervention. These findings align with previous research conducted on adult populations (Vilsbøll et al., 2012; Eng et al., 2014; Su et al., 2016; Afshin et al., 2017). A small number of headaches and injection site reactions were also reported, but there was no significant difference between the two groups, in keeping with studies in adult patients (Zaccardi et al., 2016).

Our research findings indicate that a greater percentage of participants achieved weight loss and reduction in BMI with EX10BID compared to EX2QW, aligning with the outcomes observed in previous meta-analyses (Wang et al., 2019). Therefore, the findings indicated a possible correlation between drug dosage and weight loss. It was unexpected that there was no significant decrease in HbA1c levels among obese children. This might reflect the lower pharmacokinetic effect of EX2QW than EX10BID, as only one study with EX10BID was included in HbA1c analysis. A similar pattern was seen in FDP change. Furthermore, one study with EX2QW described that adjusted estimated treatment difference in percent change in HbA1c was 0 (95% CI: −0.1 to 0.1), and in FPG was 0.5 (95% CI: −0.26–3.6), perhaps this is due to the fact that this study focues on eight-loss maintenance in adolescents with severe obesity design, high baseline BMI (39.4 ± 4.9 kg/m^2^) and low baseline FDP (78.5 ± 10.9 mg/dL) were the characteristics of participants in the exenatide-treated group enrolled in this study (Fox et al., 2022). In adults, weight loss may be greater in those with obesity-only (−2.85 and −4.47 kg) than in those with T2DM (−2.49 and −1.4 to −1.6 kg) (Chadda et al., 2021). EX2QW, the extended-release version of exenatide, has demonstrated efficacy and safety in individuals diagnosed with T2DM, offering enhanced convenience and improved patient adherence (Guo, 2016), resulting in a comparable reduction in body weight as that observed with EX10BID (Brunton and Davidson, 2016). In our investigation, EX10BID exhibited a notable advantageous impact on the management of body weight and alterations in BMI. This finding aligns with the meta-analysis conducted by Na Su (Su et al., 2016), both EX20BID and EX10BID were found to have a significant positive impact on weight loss in comparison to the placebo. However, this meta-analysis did not find sufficient evidence to support the use of once-weekly exenatide for non-diabetic individuals. Different clinical trials have targeted different patients, and more RCTs are needed in future to validate different doses of exenatide in non-diabetic obese and diabetic patients.

To our current knowledge, this research marks the first-ever meta-analysis performed to evaluate the efficacy and safety of exenatide in children diagnosed with obesity.

It is important to acknowledge certain constraints associated with this meta-analysis. First, differences in study design, baseline obesity severity and treatment dose may contribute to heterogeneity in the meta-analysis outcomes. Furthermore, the inclusion of a mere five RCTs was constrained by the small size of the samples. Finally, a cost analysis was not addressed by this systematic review and meta-analysis because there was insufficient data.

In conclusion, our comprehensive analysis revealed notable advantages of exenatide in obese children. Nevertheless, the existing literature was constrained by potential bias and a limited number of participants. More RCTs are needed in the future to confirm the efficacy and safety of this drug in treating pediatric obesity.

## Data Availability

The original contributions presented in the study are included in the article/Supplementary Material, further inquiries can be directed to the corresponding author.
