# Supplementary material for: Exenatide for obesity in children and adolescents: Systematic review and meta-analysis
Source: Front Pharmacol. 2024 Apr 3;15:1290184. doi: 10.3389/fphar.2024.1290184 (PMC11022205; doi:10.3389/fphar.2024.1290184)

**Figure S1.** PubMed search history.


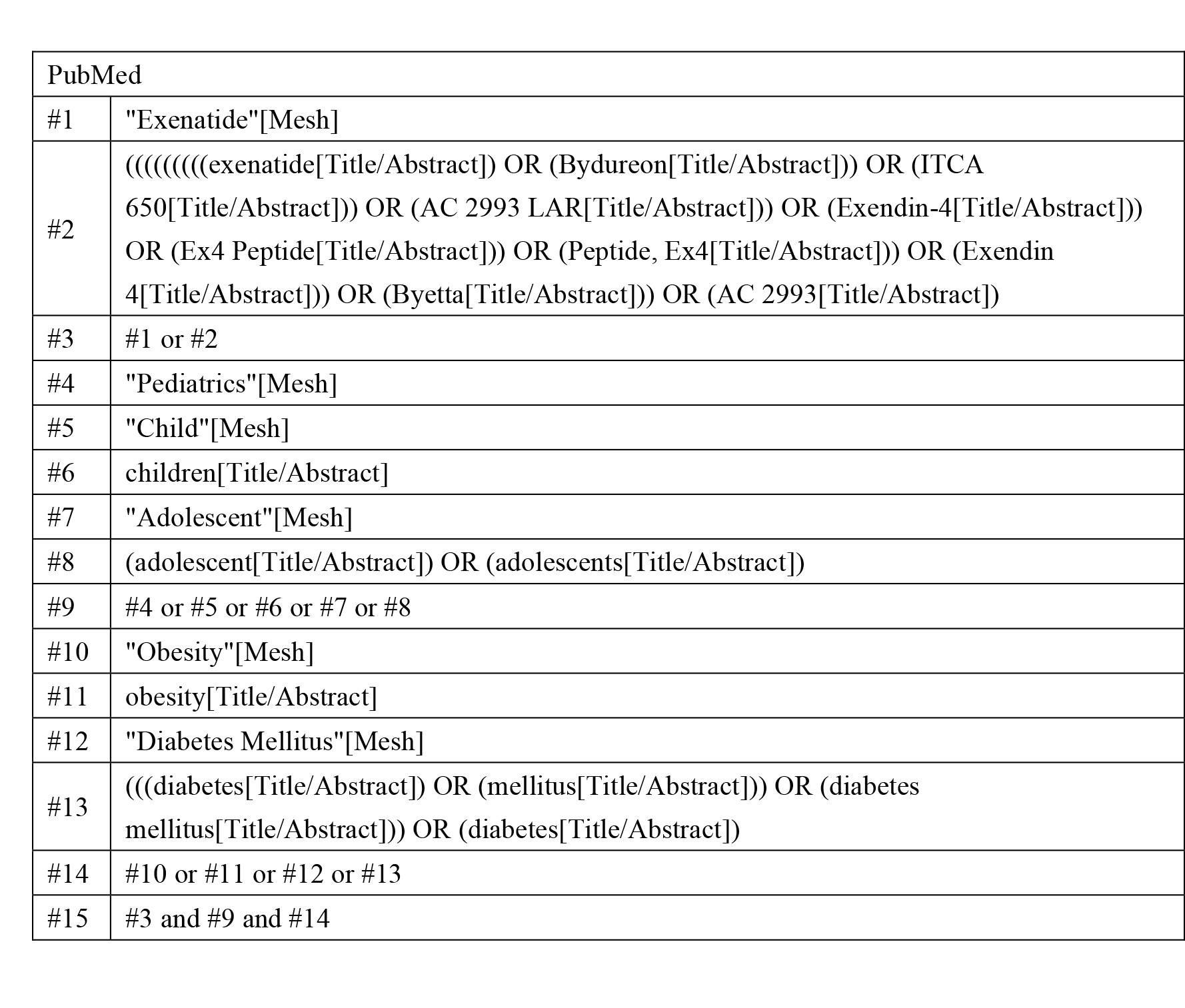


**Figure S2.** Sensitivity analysis of BMI.


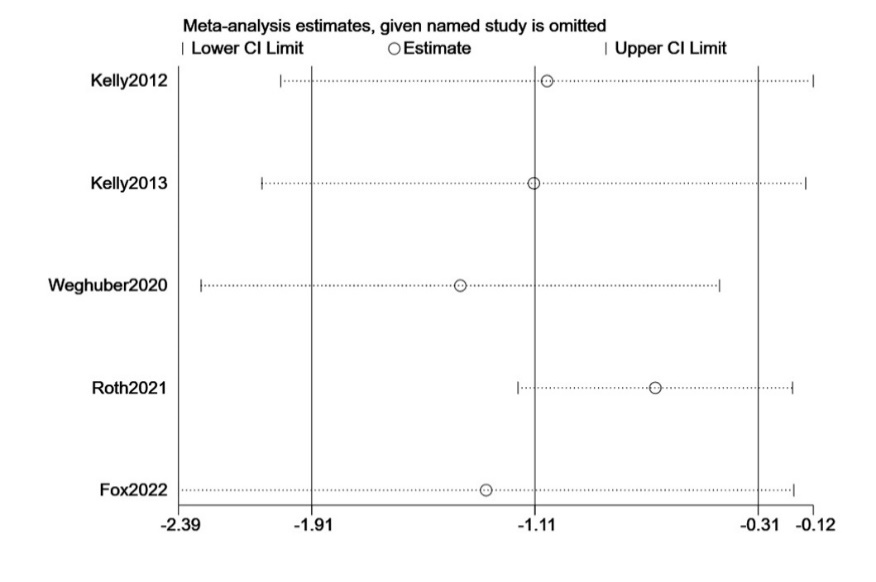


**Figure S3.** Sensitivity analysis of HbA1c.


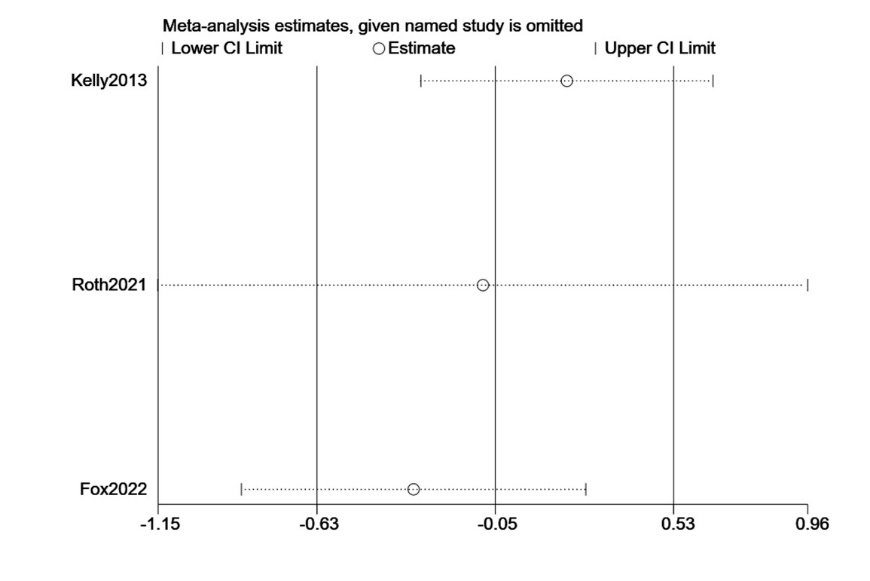


**Figure S4.** Subgroup analysis of BMI based on baseline body weight.


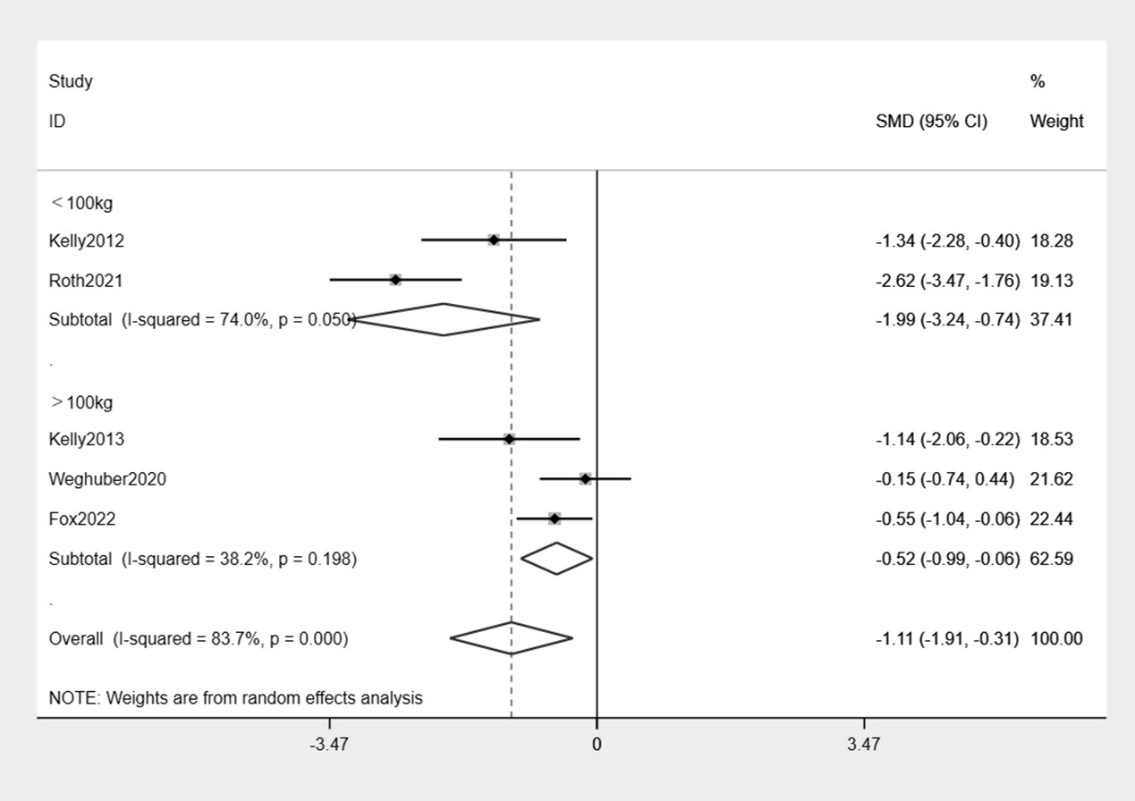


**Figure S5.** Subgroup analysis of BMI based on baseline BMI.


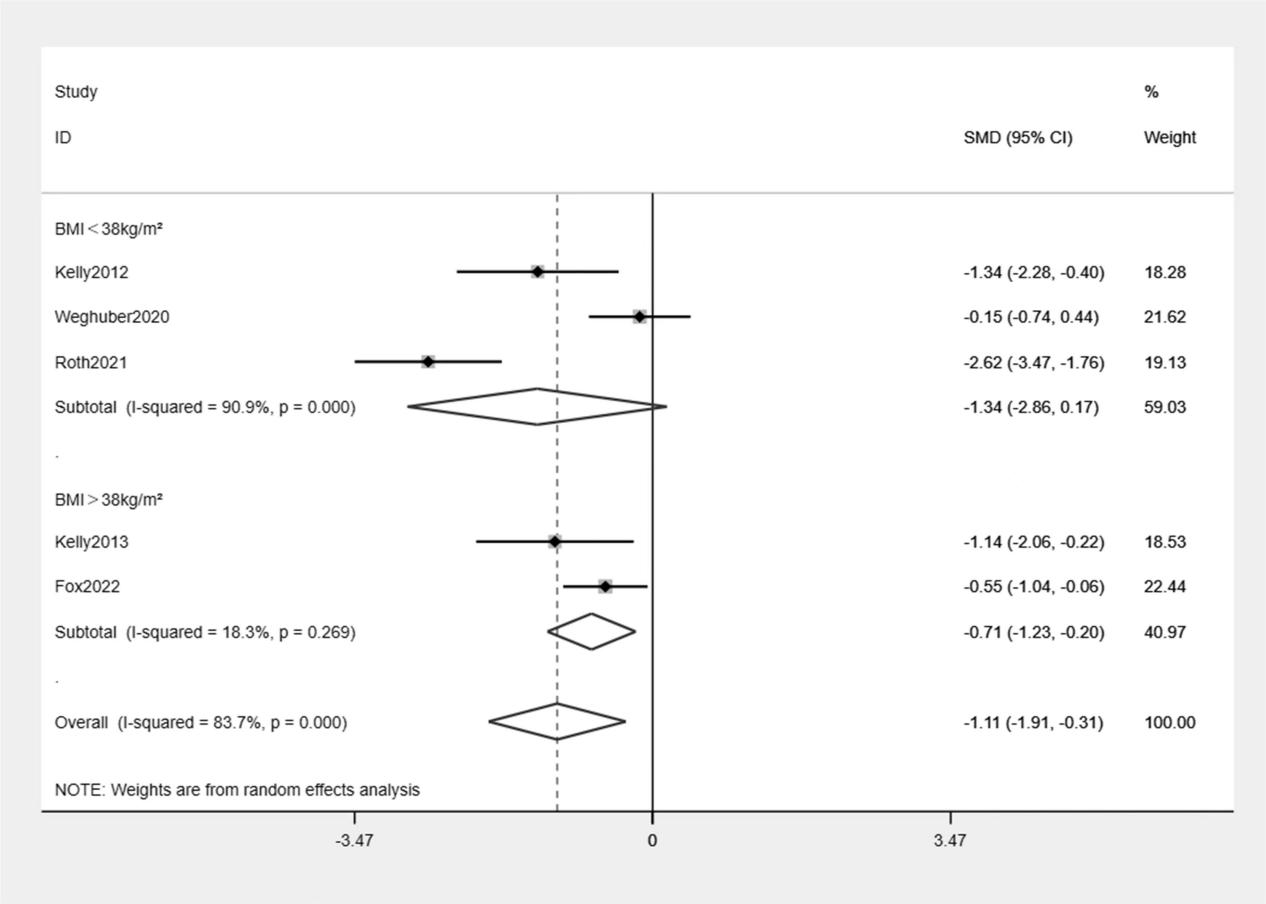


**Figure S6.** Subgroup analysis of BMI based on obesity type.


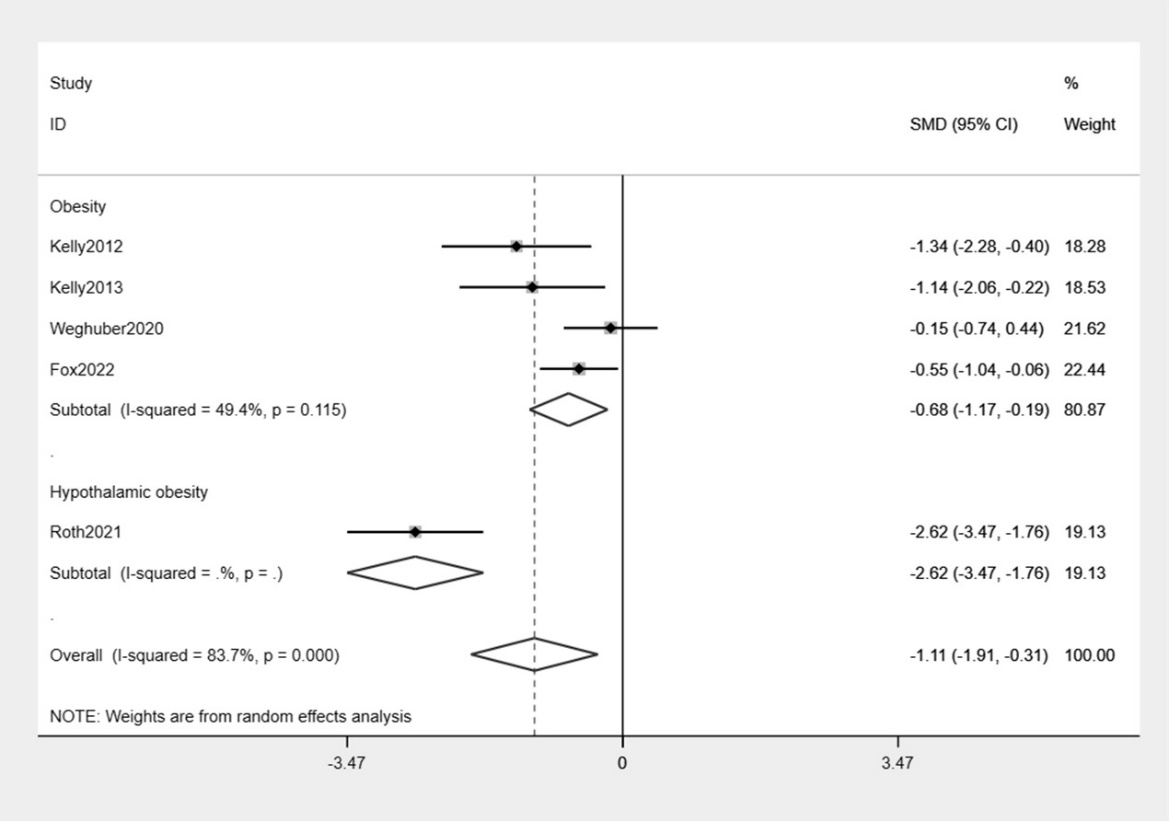


**Figure S7.** Subgroup analysis of BMI based on treat does.


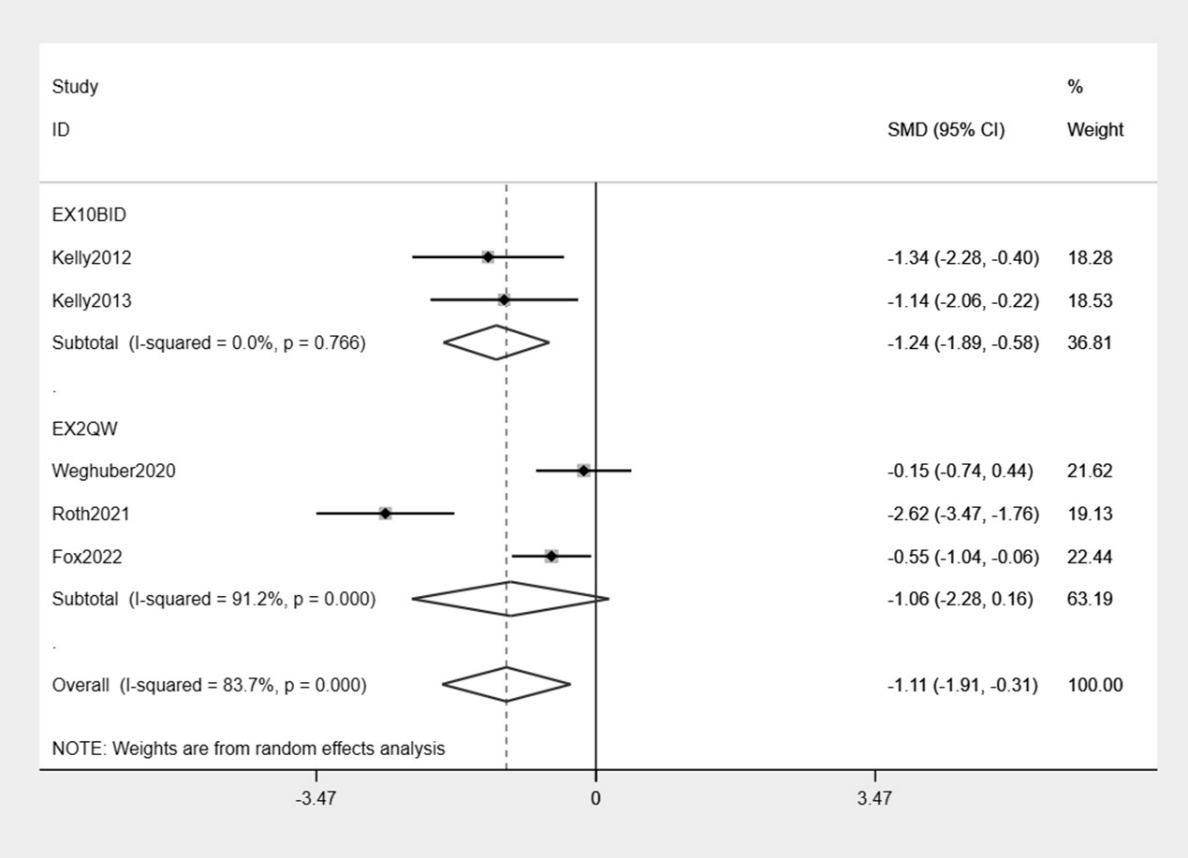


**Figure S8.** Subgroup analysis of body weight based on baseline body weight.


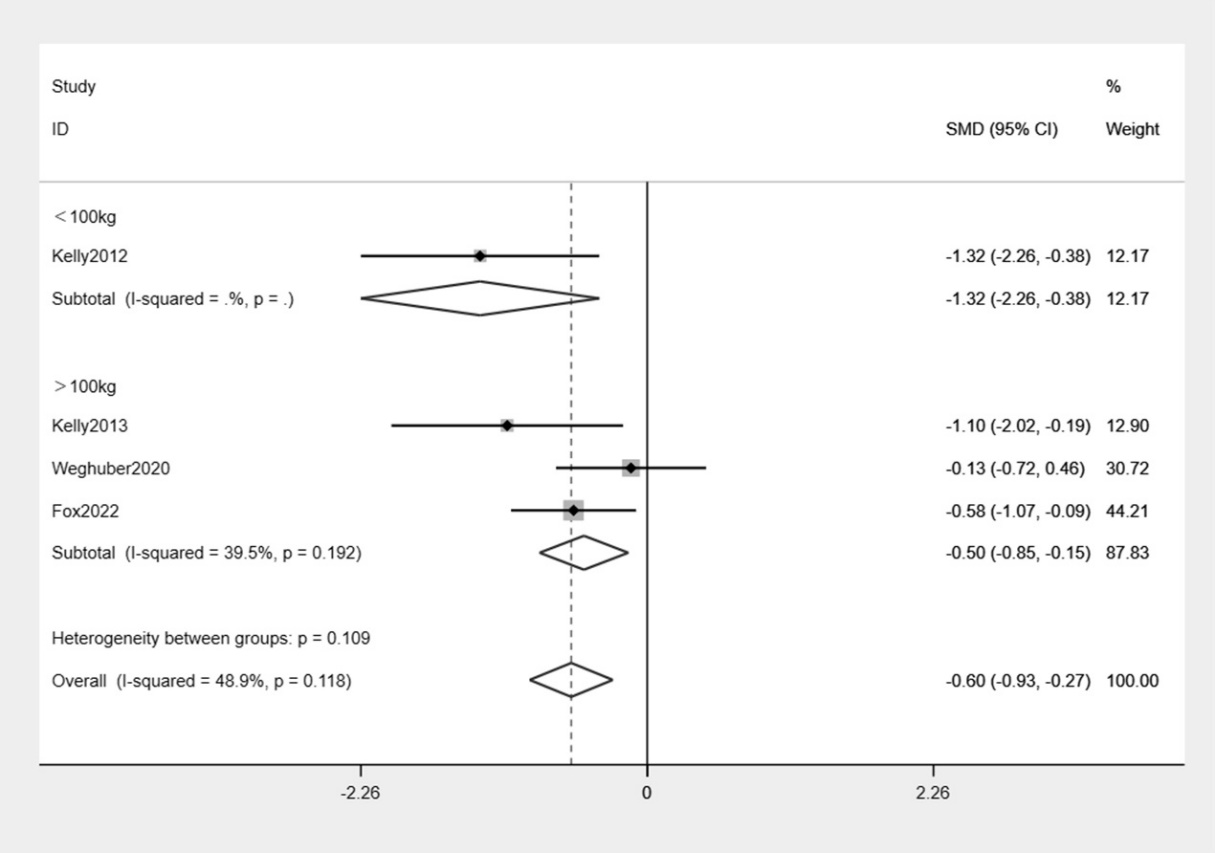


**Figure S9.** Subgroup analysis of body weight based on baseline BMI.


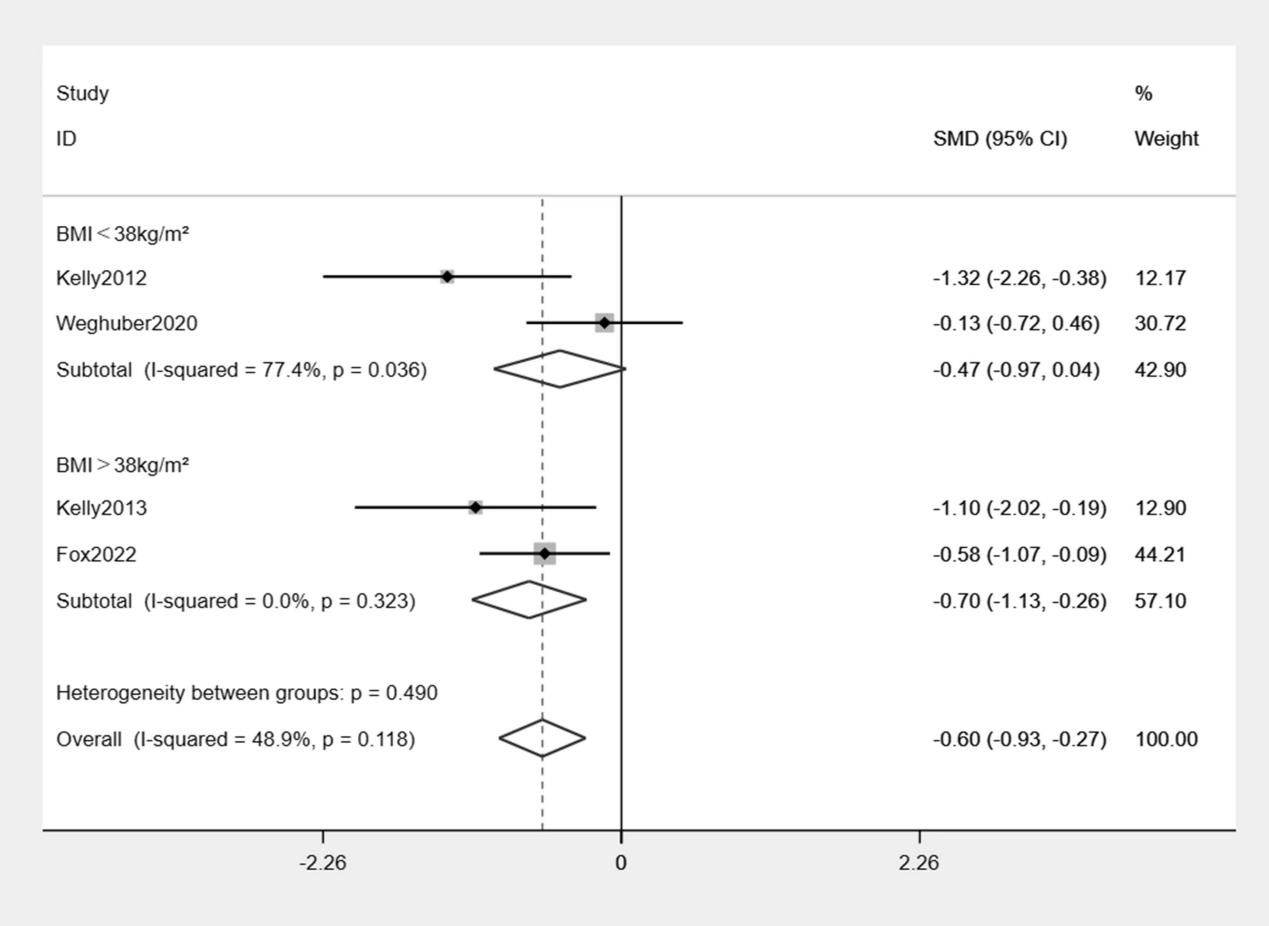


**Figure S10.** Subgroup analysis of body weight based on treat does.


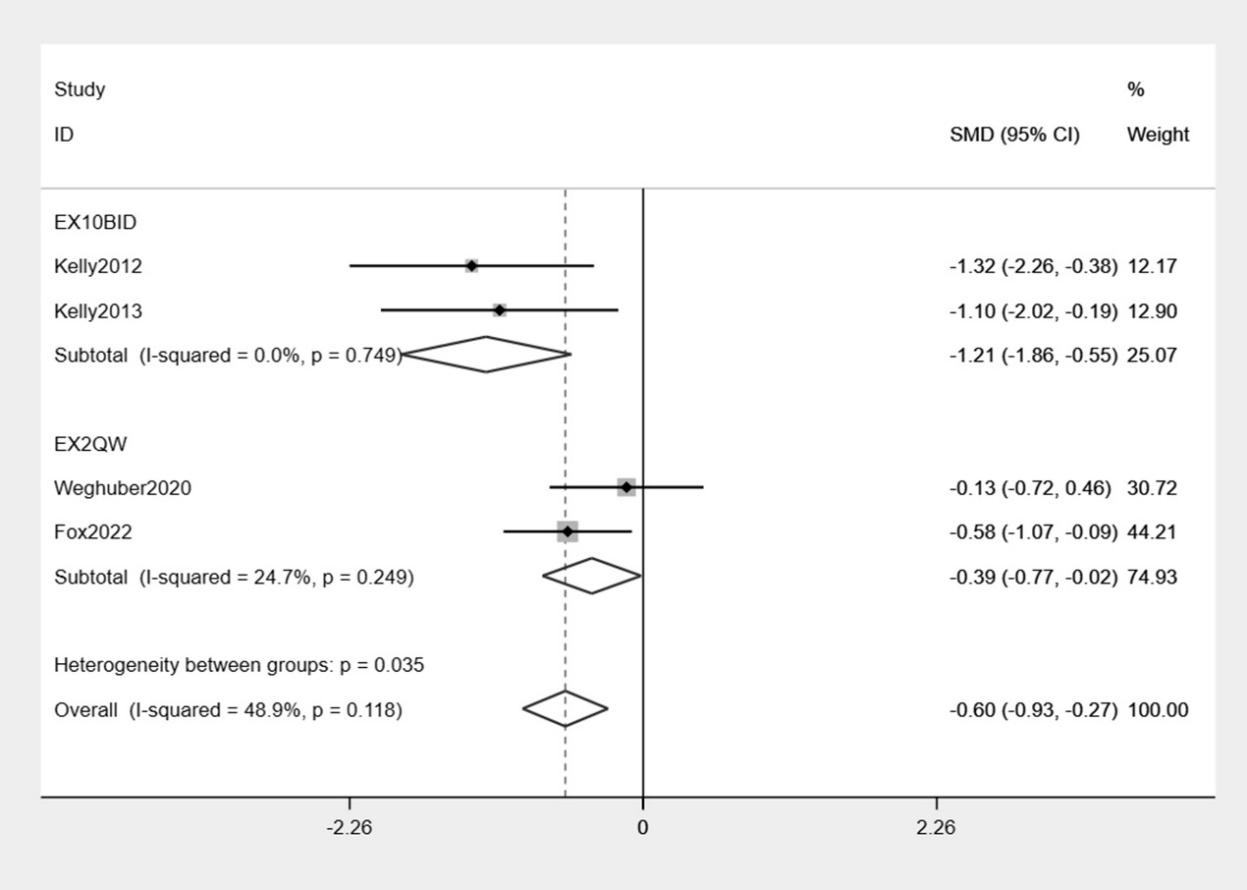

Supplement: Supplementary file 1 [file DataSheet1.docx]
